# Research on PTSD prevalence in OEF/OIF Veterans: expanding investigation of demographic variables

**DOI:** 10.3402/ejpt.v6.27322

**Published:** 2015-05-12

**Authors:** Lynnette A. Averill, CJ Eubanks Fleming, Pamela L. Holens, Sadie E. Larsen

**Affiliations:** 1 Clinical Neurosciences Division, National Center for Posttraumatic Stress Disorder, VA Connecticut Healthcare System, West Haven, CT, USA; 2 Department of Psychiatry, Yale University School of Medicine, New Haven, CT, USA; 3 Department of Psychiatry and Behavioral Sciences, Duke University Medical Center, Durham, NC, USA; 4 Department of Clinical Health Psychology, College of Medicine, Faculty of Health Sciences, University of Manitoba, Winnipeg, MB, Canada; 5 Operational Stress Injury Clinic, Deer Lodge Centre, Winnipeg, MB, Canada; 6 Psychology Service, Clement J. Zablocki VA Medical Center, Milwaukee, WI, USA; 7 Department of Psychiatry and Behavioral Medicine, Medical College of Wisconsin, Milwaukee, WI, USA

**Keywords:** PTSD, military Veteran, OEF/OIF, prevalence rates, demographics, VA service use, marital status, relationships, Don't Ask, Don't Tell, LGBT

## Abstract

**Background:**

A series of recent articles has reported on well-designed studies examining base rates of posttraumatic stress disorder (PTSD) screenings within the Operation Enduring Freedom (Afghanistan conflict)/Operation Iraqi Freedom (Iraq conflict) (OEF/OIF) military population. Although these studies have a number of strengths, this line of research points out several key areas in need of further examination.

**Objective:**

Many OEF/OIF Veterans do not use available Veterans Affairs (VA) services, especially mental health care. This highlights the need to understand the differences between those who use and do not use the VA, especially as research with pre-OEF/OIF Veterans suggests that these two groups differ in significant ways. The high rates of PTSD-related concerns in non-VA users also points to a need to understand whether—and where—Veterans are seeking care outside the VA and the accessibility of evidence-based, trauma-focused treatments in the community and private sectors. Careful examination of relationship status is also paramount as little research has examined relationship status or other relationship context issues. Social support, especially from a spouse, can buffer the development of PTSD; however, relationship discord has the potential to greatly exacerbate PTSD symptomatology. Furthermore, given the additional risk factors for sexual minority Veterans to be exposed to trauma, the 2011 repeal of the US Military “Don't Ask, Don't Tell” policy, and the emergence of the VA as likely the largest health care provider for sexual minority Veterans, it will be critically important to study the trauma and mental health experiences of this group.

**Conclusions:**

Studies that examine prevalence rates of PTSD in the returning cohort contribute significantly to our understanding of the US OEF/OIF military population. Further study of PTSD in relation to demographic variables such as VA and non-VA use, relationship status, and sexual orientation will provide rich data that will enhance our ability to develop policy and practice to provide the best care to this population.

A 2014 special issue of this journal focused on posttraumatic stress disorder (PTSD) in the military, highlighting considerable variability in PTSD prevalence rates in epidemiological studies from militaries across the world (Castro, 2014; Hunt, Wessely, Jones, Rona, & Greenberg, 2014; Taal, Vermetten, Digna, Van Schaik, & Leenstra, 2014; Van Hooff et al., 2014; Zamorski & Boulos, 2014). Indeed, several well-designed studies have recently examined the important issue of base rates of PTSD screenings specifically within the Operation Enduring Freedom (Afghanistan conflict)/Operation Iraqi Freedom (Iraq conflict) (OEF/OIF) military population (Dursa, Reinhard, Barth, & Schneiderman, 2014; Kok, Herrell, Thomas, & Hoge, 2012). This is a very important topic given both the burgeoning rates of suicide among this population (Shane, 2014) and the recent US media focus on care provided within the US Veterans Affairs (VA) system. Existing studies such as those highlighted above have examined the development of PTSD related to several key demographic variables (i.e., age, gender, employment/return to work status, race, and military service variables). Although these studies have a number of strengths, this line of research points out several key areas in need of further examination. We suggest a need for additional study focusing on the following demographic variables in relationship to PTSD in US OEF/OIF Veterans: VA status (with a particular focus on non-VA users), relationship status, and sexual orientation.

Although the studies by Dursa et al. and a few others have included both Veterans who use and do not use VA health care, many studies of PTSD prevalence in US Veterans have exclusively focused on Veterans using VA health care. However, many OEF/OIF Veterans do not use available VA services, especially mental health care (e.g., DeViva, 2014; Elbogen et al., 2013). This points to a need to understand the differences between those who use or do not use the VA. Research with pre-OEF/OIF Veterans has shown that these two groups differed in significant ways, including illness level. Indeed, in the Dursa et al. sample, more VA users screened positive for PTSD; yet, the rate of positive screens in the sample of non-VA users was also high, indicating a need for mental health care in both groups.

Several researchers have started to address how to engage OEF/OIF Veterans in VA care and have shown the utility of using brief motivational interviewing interventions (Seal et al., 2011; Stecker, Fortney, & Sherbourne, 2011), providing services through telehealth to improve access (Tuerk, Yoder, Ruggiero, Gros, & Acierno, 2010), or integrating mental health assessment into primary care intakes (Batten & Pollack, 2008; Seal et al., 2011). Given the high stigma around mental health care among younger Veterans, and the high likelihood of these individuals raising mental health concerns in primary care rather than in mental health appointments (Seal et al., 2011), such interventions hold promise and merit further investigation.

The high rate of PTSD-related concerns in non-VA users also points to a need for research to understand whether—and where—Veterans are seeking care outside the VA. Only about 60% of returning Veterans are engaged in VA care (Veterans Health Administration [VHA], 2014), leaving a high number of Veterans in the community with potentially untreated PTSD and other mental health concerns. Unfortunately, because most research on Veterans only addresses the population seeking VA treatment, we do not know much about those who do not use VA services. There has been an appropriately strong emphasis on standardized rollouts of trauma-focused, evidence-based psychotherapies (EBPs) for PTSD within the VA (Karlin et al., 2010), but resources for EBPs outside of VA appear to be more limited. Non-VA clinicians are less likely to have access to training in military culture and to the standard EBP training and consultation programs available through the VA. In addition, some alternative treatments have been developed to address high drop-out rates for trauma-focused treatments, but these are scarce both in and out of the VA system (e.g., Markowitz et al., 2015). Programs such as Welcome Back Veterans are working with community organizations to more widely disseminate appropriate resources, and to encourage VA/community collaboration. Some treatment packages, such as Cognitive Processing Therapy (Resick & Schnicke, 1992), have created online training modules to allow for further distribution of treatment protocols. Given that many Veterans never access the VA services, and also that many Veterans are concerned about seeing outside providers for fear that they will not understand military-related concerns (Sayer et al., 2009), it will be critical to disseminate and track access to such treatments for Veterans in the community.

In addition to studying VA status and looking more closely at non-VA users, it would be useful to examine two further demographic factors in future studies of PTSD prevalence in the OEF/OIF populations: relationship status and sexual orientation. Regarding relationship status, research suggests that social support, especially support from a spouse, can buffer the development of PTSD in trauma survivors in general and Veterans in particular (Dinenberg, McCaslin, Bates, & Cohen, 2014; Evans, Steel, Watkins, & DiLillo, 2014; Vogt et al., 2011). Conversely, relationship discord has the potential to greatly exacerbate PTSD symptomatology (Evans, Cowlishaw, Forbes, Parslow, & Lewis, 2010). Divorce rates among military couples increased by almost 50% over the first decade of the OEF/OIF era (Military One Source, 2011), suggesting that marital distress in military/Veteran couples is alarmingly high, and is likely playing a role in returning Veterans developing PTSD symptoms.

Relatively, few prevalence studies of PTSD in the OEF/OIF populations have examined the association between relationship status and mental health, and those that have have found mixed results. For example, some studies have found no relationship between marital status and PTSD (Riddle et al., 2007; Schell & Marshall, 2008), one suggested that marital status increases risk for development of PTSD (Grieger et al., 2006) and another found higher odds of new-onset PTSD (but not persistent PTSD) among never married or divorced personnel (Smith et al., 2008). It would be useful to go beyond the categorical marital status to investigate the potentially critical moderating role of marital/committed relationship quality. For instance, Vogt et al. (2011) found that the presence of post-deployment stressors (e.g., “gone through a divorce or been left by a partner”) had a direct effect on the development of posttraumatic stress symptoms for both men and women. Similarly, Vasterling et al. (2010) found that home front concerns (e.g., “the well-being of my family”), along with post-deployment stressors, collectively contributed to significant variance in PTSD change scores from pre- to post-deployment. Thus, it will be essential to examine the role of relationship status and quality in the development and prevalence of PTSD in the current returning cohort.

Finally, given the 2011 repeal of the US Military “Don't Ask, Don't Tell” policy as well as the emergence of the VA as likely the largest health care provider for sexual minority Veterans (Mattocks et al., 2013), it will be important to study the trauma and mental health experiences of lesbian, gay, bisexual, and transgender (LGBT) Veterans. One estimate is that LGBT individuals account for 2.2% of all military personnel, a number that is only likely to increase (Gates, 2010). There have been very few studies on LGBT mental health in the military and in Veteran populations (e.g., Sherman et al., 2014), and even fewer focusing on OEF/OIF Veterans. Initial evidence suggests that LGBT service members endure higher rates of sexual trauma, both before and during their military experiences (Mattocks et al., 2013), and that LGBT Veterans may be disproportionately targeted for sexual victimization (Kauth, Meier, & Latini, 2014). Furthermore, there is some evidence that anxiety around concealment of one's sexual orientation in service is correlated to later depression and PTSD symptoms (Cochran, Balsam, Flentje, Malte, & Simpson, 2013). Although there is no longer an official mandate to conceal one's orientation, norms within the military may still encourage such concealment. As the VA starts to provide treatment to LGBT Veterans, further research on the prevalence and consequences of trauma and PTSD—and their interaction with sexual orientation norms and changing laws—within this population will be invaluable.

Studies that examine prevalence rates of PTSD in the returning cohort contribute significantly to our understanding of the US OEF/OIF military population. Although beyond the scope of this paper, there are likely additional variables that influence the prevalence rates for PTSD in the current cohort of Veterans. For example, concerns have been raised about potential fabrication of PTSD symptoms related to financial incentives within the VA, an issue worthy of careful study (e.g., see Constans et al., 2014). It is also important to note that we focus in this manuscript on a US OEF/OIF population. One meta-analysis has shown that base rates of PTSD are similar across US and UK samples, though post-deployment rates are higher in the US samples, possibly due to higher combat exposure, longer deployments, and shorter recovery periods between deployments (Kok et al., 2012). The demographic variables we highlight may differ across countries, based on variability in post-deployment debriefing and decompression practices, military culture regarding the psychiatric consequences of deployment, military and cultural/societal attitudes regarding sexual orientation and identity, health care systems, policies, and so on. Overall, we believe that further study of PTSD in relation to demographic variables such as VA service utilization, relationship status, and sexual orientation will provide rich data that will further our ability to develop policy and practice to provide the best care to this population across cultures.
